# Assembly of Soft Electrodes and Ion Exchange Membranes for Capacitive Deionization

**DOI:** 10.3390/polym11101556

**Published:** 2019-09-25

**Authors:** Silvia Ahualli, Sergio Orozco-Barrera, María del Mar Fernández, Ángel V. Delgado, Guillermo R. Iglesias

**Affiliations:** 1 Department of Applied Physics, Faculty of Science, University of Granada, 18071 Granada, Spain; wakthor97@correo.ugr.es (S.O.-B.); adelgado@ugr.es (Á.V.D.); 2 Department of Fluidic and Energy Science, Central America University, (01)168 San Salvador, El Salvador; mdelmarf@ugr.es

**Keywords:** capacitive deionization, polyelectrolytes, salt adsorption, soft electrodes, ion exchange membranes

## Abstract

The responsible use of water, as well as its reuse and purification, has been a major problem for decades now. In this work, we study a method for adsorbing ions from aqueous solutions on charged interfaces using highly porous electrodes. This water purification process is based on the electric double layer concept, using the method known as capacitive deionization (CDI): If we pump salty solutions through the volume comprised between two porous electrodes while applying a potential difference to them, ions present in the solution are partially removed and trapped on the electrode surfaces. It has been well established that the use of carbon electrodes in combination with ion exchange membranes (membrane-CDI) improves the efficiency of the method above that achieved with bare activated carbon. Another approach that has been tested is based on coating the carbon with polyelectrolyte layers, converting them into “soft electrodes” (SEs). Here we investigate the improvement found when combining SEs with membranes, and it is shown that the amount of ions adsorbed and the ratio between ions removed and electrons transported reach superior values, also associated with a faster kinetics of the process. The method is applied to the partial desalination of up to 100 mM NaCl solutions, something hardly achievable with bare or membrane-covered electrodes. A theoretical model is presented for the ion transport in the presence of both the membrane and the polyelectrolyte coating.

## 1. Introduction

In 1960 a pioneering work on the concept of water desalination was reported by Blair, Murphy, and co-workers [1] under the name of “electrochemical demineralization of water”. In recent years, and returning to this original idea, the method known as capacitive deionization (CDI) emerged as an energy-effective alternative to other conventional methods. This process partially removes the ions present in solution by taking advantage of the electric double layer (EDL) properties. The technique is based on the application of an electric potential difference between a pair of porous electrodes, through which the solution to be desalinated is pumped. In the process, ions migrate to the oppositely charged electrode, forming the electric double layer on the interface, until totally screening the charge at the electrode surface. The charged surface and its EDL constitute a sort of capacitor, hence the name of “capacitive” given to this method. Furthermore, the specific capacitance of the EDL can be very large, and a sufficient amount of interfacial area is needed for significant storage of ions. The overall process is optimum when desalinating solutions with concentrations under 10 g/L.

These arguments have boosted the investigation of CDI methods, focusing mainly on their implementation at a scale beyond the laboratory bench and the search for materials specifically chosen for improving the efficiency of the process. Among them, activated carbon is probably the most largely used [2] as a consequence of its high surface area and good conductivity, but other carbon-based materials have been proposed [3,4]. Promising results have shown that graphene aerogel or hydrogels, alone or in combination with single- or multi-walled carbon nanotubes, can enhance considerably the salt adsorption capacity by controlling the interlayer spacing of graphene and its internal resistance [5,6]. Furthermore, the combination of bare carbon electrodes and ion-exchange membranes has demonstrated excellent desalination results, certainly improving on bare carbon [7,8,9]. The improvement is possible because the presence of a cation-exchange membrane on the negative electrode blocks the passage of anions from this into the solution; the same applies to cations on the positive electrode. The method, known as membrane-based capacitive deionization (or just membrane capacitive deionization, MCDI) inspires the alternative here proposed. This is based upon depositing a layer of polyelectrolyte (PE) (cationic or anionic, depending on the polarity of the membrane) on the carbon active substrate before placing the respective membrane.

Previous results have demonstrated that the PE layer coats most of the carbon surfaces, mainly the macropores [10,11,12]. Because of the similarity of the resulting interface to that acquired by colloidal particles coated by the same procedure, the resulting electrodes are known as soft electrodes (SEs). Although the overall performance may be lower than that of membrane-based methods (both have been compared regarding capacitive energy harvesting), the lower internal resistance [13], as well as the lower price and ease of preparation, points to SEs as a plausible or complementary approach. It is worth mentioning that this is not the only method that has been proposed based on electrode modification. A very promising one has been denominated inverted CDI (i-CDI), and involves a chemical modification of the electrodes so that a spontaneously formed EDL works in the adsorption stage, while the external voltage is only applied in the desorption one [14].

The approach followed in the present work includes the desalination of NaCl solutions using bare, membrane-coated, and PE-coated electrodes, comparing their respective performances. We also study the improvement that can be reached by the combination of membranes with SEs, a method that can be called SE-MCDI. In additions to the electrode microstructure, the operational routines have been found to play a fundamental role. These will also be explored here by considering different methods, including short-circuiting the electrodes vs. applying opposite potentials during the ion adsorption and releasing steps. Several NaCl concentrations in the solutions to be desalinated are tested. The theoretical model previously elaborated by Biesheuvel et al. for MCDI [15] will be modified for the case of SE-CDI + MCDI, in order to gain insight into the mechanisms responsible for the desalination behavior of soft electrodes combined with membranes.

## 2. Experimental

### 2.1. Materials

For the experimental work, 10, 40, and 100 mM NaCl (Sigma Aldrich, Darmstadt, Germany) solutions were prepared. A Milli-Q Academic System (Millipore, Darmstadt, Germany,) was used to deionize and filter the water.

In order to prepare the electrodes, SR-23 carbon particles (from MAST Carbon International Ltd. (Birmingham, UK)) were employed. This is an ideal material due to its large surface area (959 m^2^/g) and its proper fraction of mesopores, which provide suitable ion reservoirs in the adsorption stage.

Poly-(diallyldimethylammonium chloride) (PDADMAC with a typical molecular weight of 100,000 g/mol) and poly(sodium 4-styrenesulfonate) (PSS with a typical molecular weight of 70,000 g/mol) aqueous solutions were prepared for the polyelectrolyte coatings, cationic and anionic, respectively, of the carbon electrodes. The polyelectrolytes were purchased from Sigma Aldrich. The anion exchange membrane, AMX, and the cation exchange one, CMX, (thickness *L_mem_* = 140 µm and *L_mem_* = 170 µm, respectively) were purchased from Neosepta (Tokuyama, Tokyo, Japan).

**Figure 1:**
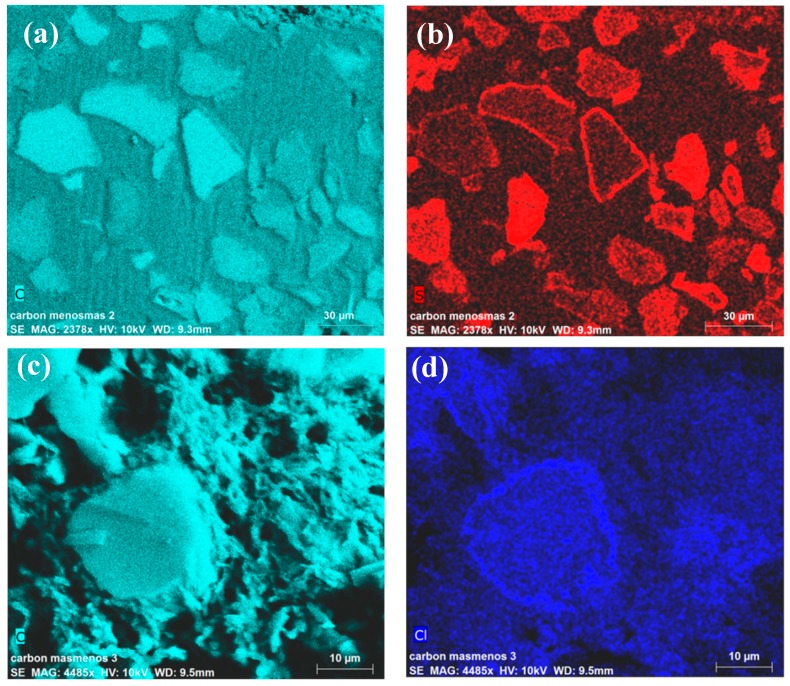
Carbon (**a**,**c**), sulfur (**b**), and chlorine (**d**) maps obtained by EDX for (top row) PSS- and (bottom) PDADMAC-coated electrodes.

The surface composition of the coated carbons was determined using EDX spectroscopy with a FEI Environmental Scanning Microscope Model Quanta 400 (Hillsboro, OR, USA), using chlorine as a test of the presence of PDADMAC and sulfur for detecting the adsorption of PSS. Samples were previously lyophilized and fixed in resin, and afterward cut in a Leica Ultracut R microtome (Wetzlar, Germany) and polished. Figure 1 illustrates some results. Note how the arrangements of sulfur and chlorine indicate that the polyelectrolytes coat the particles with a thin layer, visible as a colored line surrounding the sliced carbon. It is also apparent that the polyelectrolytes are also located inside the particles, lining the pore walls.

### 2.2. Setup

The cell consisted of two planar, rectangular electrodes facing each other at a distance of 500 µm, separated by a rubber ring. Each electrode was based on a graphite current collector (10 cm long and 5 cm wide) onto which was deposited a layer of a suspension containing 3 g of activated carbon particles and 10 g of a 33 g/L solution of poly(vinylidene-fluoride) (PVDF, manufactured by Arkema, (Colombes, France) as Kynar HSV 900, with molecular weight approximately 1,000,000) in 1-methyl 2-pyrrolidone (Sigma Aldrich, Darmstadt, Germany). In order to fix the carbon suspension onto the graphite collectors, they were dried during 24 h at 100 °C. The polymer coating on the electrode surfaces was carried out by immersing each electrode in a 100 mM (on a monomer basis) aqueous solution of its corresponding polyelectrolyte (PSS for the anode, and PDADMAC for the cathode). This process was extended during 24 h under constant magnetic stirring of the polyelectrolyte solutions, and the electrodes were then rinsed with water. Finally, ion-exchange membranes were placed close to each electrode (the cation(anion) exchanger on the PSS(PDADMAC)-coated one) and fixed by means of a rubber separator.

During the desalination process, the solution was pumped through the cell from a storage vessel. The conductivity and temperature of the solution and temperature were measured at the exit of the cell, using a probe (529670, Leybold, Dresden, Germany) read by a Leybold 524D10 Cassy Lab interface. Simultaneously, the voltage applied to the system and the current were directly measured from the power source. The exit solution was sent back to the storage vessel, which had a volume large enough to ensure that the inlet conductivity did not change appreciably between successive cycles.

Each capacitive deionization cycle consisted of two steps:

Adsorption: The electrodes were electrically powered by applying a constant voltage (CV); this drove the ions from the incoming solution into the macropores of the oppositely charged electrode.

**Figure 2:**
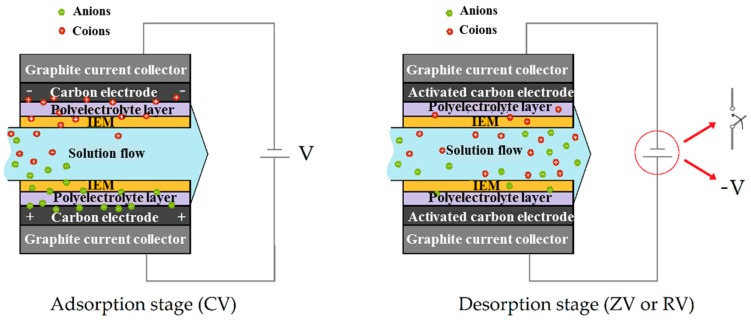
Schematic representation of adsorption step (**left**) by connecting a constant voltage (CV) power source. The desorption stage (**right**) proceeds by either short-circuiting the system (ZV—zero voltage) or applying the opposite potential difference (RV—reverse voltage).

Desorption: Once the ions neutralized the electronic charge, it was necessary to drain the macropores, allowing the reuse of the system for another adsorption step. Desorption was achieved by setting either an external short circuit (zero voltage, ZV) or a reversing the applied voltage (RV). Constant voltages of values 0.9 and 1.2 V were used for all the measurements. The stages mentioned are illustrated in Figure 2.

## 3. Results and Discussion

### 3.1. Effect of the Applied Field Strength

In order to compare the effect of the combined use of the polyelectrolyte layer and ion exchange membranes, four different assemblies of the CDI cell were used:

1. Bare activated carbon electrodes (Bare)
2. Polyelectrolyte-coated electrodes, or soft electrodes (SEs)
3. Ion exchange membranes in contact with the electrodes (MCDI)
4. Combination of polyelectrolyte coating with the use of membranes (SE + MCDI)

**Figure 3:**
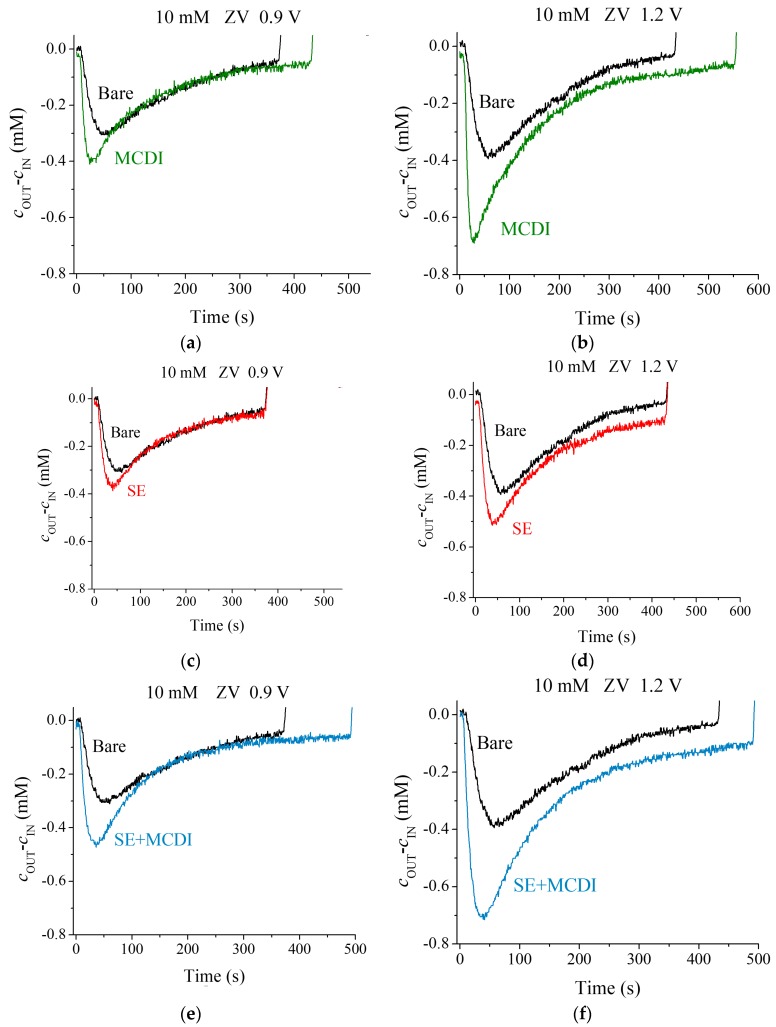
Solution concentration difference between the exit and input of the cell for applied voltages of 0.9 and 1.2 V, and a 10 mM NaCl feed solution. Comparison between bare carbon electrodes with membrane capacitive deionization (MCDI) (**a**,**b**), soft electrodes (SE) (**c**,**d**), and SE + MCDI electrodes ensembles (**e**,**f**). All the cycles were performed at ZV desorption.

The typical cycles of adsorption/desorption steps are represented in Figure 3 for the different cell configurations and applied voltages. The charged electrodes attract the counterions removing them from the solution and, therefore, producing a conductivity decrease. The migration of ions to the EDL continues until the charge on the electrodes is totally screened. Because the solution is continuously pumped, the measured conductivity reaches a minimum value, and it progressively returns to that of the feed solution, as the capacity of the electrodes is exhausted. This step is followed by the desorption one, which forces the previously stored ions back into the solution, producing an increase in its conductivity (a complete cycle is represented in Figure S1, Supplementary Information). It is easy to observe that by increasing the voltage a larger amount of ions is removed.

We observe in Figure 3 that both MCDI and SEs yield a better performance than simple CDI, as in fact found by other authors [16,17,18,19]. This difference comes from the coion blocking ability that membranes and polyelectrolyte coating offer: When the EDL is built, counterions are attracted and coions expelled from the micropores. The latter are blocked by the charged region (either membrane or polyelectrolyte layer), avoiding the flow from micropores to the spacer where the solution is flowing. Therefore, the coions will be stored in the macropores, attracting further counterions to guarantee the electrical neutrality of this region. In this way, the coating produces an increment of the salt adsorption and of the efficiency at the same time. Both MCDI and SE lead to an improvement in the salt adsorption compared to CDI using bare electrodes, as depicted by the larger area under the curve of concentration difference of Figure 3. Both methods combined (SE + MCDI) reveal a higher salt adsorption manifested in a deeper minimum and slower rise of the measured conductivity of the solution.

### 3.2. Zero-Voltage versus Reverse-Voltage Desorption

At the end of the adsorption step, the electrodes are discharged by either short-circuiting them (ZV) or applying a reverse voltage (RV) changing the electrodes polarity [20]. Due to electrostatic repulsion, coions (counterions in the adsorption step) are released to the spacer, increasing the concentration of the output solution. If the electrodes are neither covered with ion exchange membranes nor treated, those coions are attracted by the opposite electrode, so that an adsorption step immediately will follow the desorption peak, showing the “frequency doubling” phenomenon [20], so named because two adsorption steps occur in the whole CDI cycle, whatever the polarity of the applied voltage.

In the case of MCDI, the RV desorption step (Figure S1) differs from that corresponding to bare electrodes: The counterions (former coions) are not able to reach the opposite electrode since membranes block the entry, so that only the release of ions will take place and not the subsequent adsorption. A similar behavior is found with SE and the combination of SE + MCDI. Examples are presented in Figure S1.

### 3.3. Comparison of Different Saline Concentration Solutions

For each voltage and a 10 mM salt concentration, as depicted in Figure 3, the bare electrodes offer lower ion adsorption, followed by the polyelectrolyte coating, and finally membranes yield the highest performance of the three methods. In the light of these results, one may wonder what the behavior would be at even larger concentrations of feed solution.

**Figure 4:**
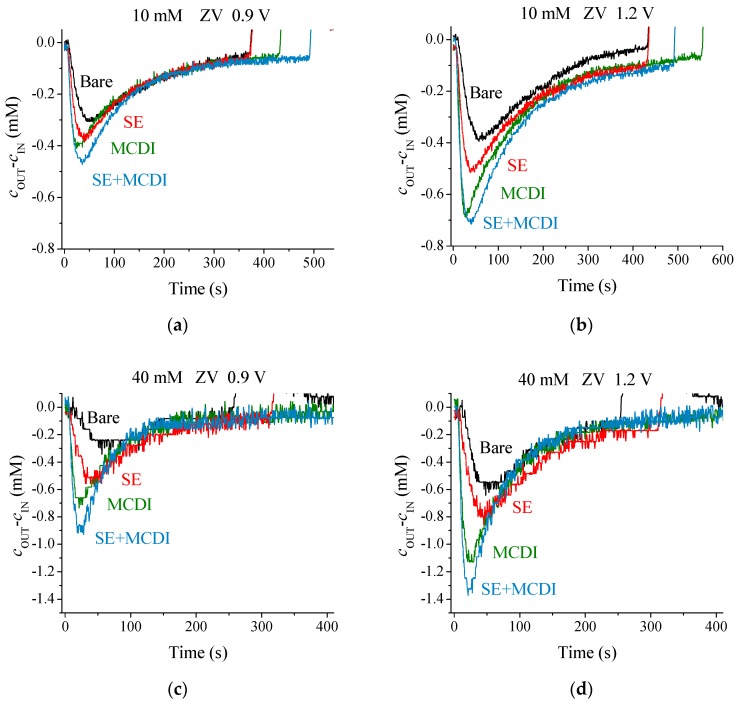
Adsorption stage for 10 and 40 mM NaCl at 0.9 and 1.2 V with ZV desorption using different techniques: (**a**) 10 mM NaCl, ZV 0.9 V; (**b**) 10 mM NaCl, ZV 1.2 V; (**c**) 40 mM NaCl, ZV 0.9 V; (**d**) 40 mM NaCl, ZV 1.2 V.

For 40 mM solutions, a similar behavior to that with 10 mM is observed, as noted in Figure 4. It is important to keep in mind that even though the amount of NaCl in the solution is increased by a factor of four, the amount of adsorbed ions is only doubled. This behavior is the consequence of the fact that the amount of adsorbed ions only depends on the electronic charge at the solid/solution interface, and the micropore volume, which will only slightly change with the solution concentration. In fact, this is one of the limitations of capacitive methods to purify concentrated ionic solutions (in the case of sea water, about 1 kg of carbon in the electrodes would be necessary to desalinate a volume of 1 liter). However, a faster kinetic response in the case of higher salt concentration, with sharper adsorption peaks for MCDI and SE-MCDI, is appreciated. This behavior, a consequence of a lower internal resistance, leads to faster cycles. Data in Figure 4 also show that the amount of salt adsorbed by SEs is lower than that achieved in MCDI. A possible explanation for this is that the PE coating (Figure 1) is not as uniform as the membrane microstructure, making the soft electrode less ion-selective than membranes. In spite of this drawback, the use of SEs should not be discarded, considering that the differences are not as large, considering the lower price and ease of implementation of SEs. Of course, best results are achieved when both membranes and polyelectrolytes are combined.

An ideal application of CDI would be desalinating solutions with even higher ionic concentrations, close to sea water. Based on this, and considering the good results obtained with the combined method in dilute solutions, it appeared interesting to face the desalination of 100 mM NaCl solutions, comparing how membranes work under these conditions and the possible improvement that can be achieved when adding the polyelectrolyte layers. 

**Figure 5:**
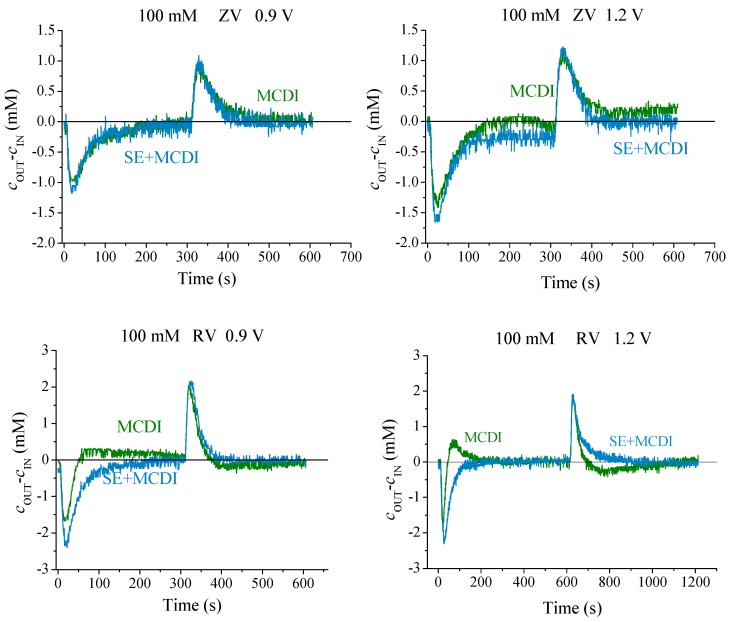
Capacitive deionization (CDI) cycles for 100 mM NaCl solutions, using MCDI and SE + MCDI, set to ZV (**top**) and RV (**bottom**) desorption mode.

The adsorption curves for MCDI under ZV desorption conditions show a different trend than that found for more dilute solutions (Figure 5). This difference is accentuated if we set RV desorption, and in fact the response turns out to be similar to that found with bare carbon electrodes (Figure S1): In each adsorption/desorption step a small peak is detected opposite to the main one, suggesting the superposition of two opposite trends for the concentration difference, a consequence of the adsorption and release of the ions with a slight time difference. The reason for this can be found in the membrane’s behavior at high ionic concentration. Due to the high amount of ions present in the solution, they do not block coions as efficiently as is expected theoretically. 

However, the combined method, SE-MCDI, allows a correct and efficient ion blocking that performs similarly to the low salt concentration case. Thus, the polymeric layers act as reinforcement for the membranes, keeping an optimum behavior. These facts can be quantified by analyzing the salt adsorption of the system, its efficiency, and the energy consumed for the process in each case. 

It is important to mention that these results might be affected by pH variations during the CDI process. Such pH changes are associated with faradaic reactions on the electrodes. As such, it can be expected that they will be minimized upon coating by the polyelectrolyte, the membrane or both. This has been demonstrated by Tang et al. [21] and is confirmed by our own data on pH contrast between ingoing and outgoing solutions. Measurements were performed for 100 mM solutions, and it was found that the maximum pH variation during the deionization step was from 5.6 to 4.9, that is, a conductivity change of 3.6 × 10^−3^ mS/cm, negligible as compared to the 0.20 mS/cm measured. These results are in agreement with the hypothesis that coating limits pH variations, and that the conductivity changes observed are not a result of the latter.

### 3.4. Salt Adsorption, Efficiency and Consumed Energy

The amount of adsorbed salt in each cycle is one of the most important quantitative results, because it measures the capacity of the system for removing ions from water. The specific salt adsorption capacity, *SAC*, is given by the mass of adsorbed ions per mass of electrodes, $m_{elec}$ [2,16]:

(1) $$SAC=n\frac{M_{salt}}{m_{elec}}$$

where *n* is the number of moles adsorbed in the cycle and *M_salt_* is the salt molecular weight. The number of adsorbed moles of ions is given by the enclosed area below the adsorption curve and is calculated as [16,20]:

(2) $$n=\int_{0}^{t_{ads}}\left(c_{out}(t)-c_{in}\right)\phi_{v}dt$$

where $\phi_{v}$ is the pumping velocity of solution in the CDI circuit and *t_ads_* is the duration of the adsorption step.

Another proposed metric of the desalination performance is the specific adsorption rate (*ASAR*), defined, for a single cell, as [22,23]

(3) $$ASAR=\frac{n}{At_{cycle}}$$

where *A* the projected area of the electrodes and *t_cycle_* the duration of the complete cycle.

**Figure 6:**
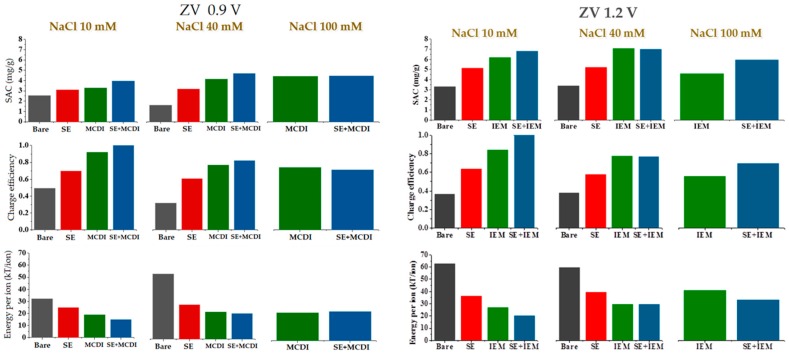
Salt adsorption, charge efficiency, and energy per ion removed calculated for electrode configurations under ZV desorption conditions for the adsorption cycle shown in Figure 4.

The *SAC* calculated using polyelectrolyte coatings, membranes, or both together, is represented for three ionic concentrations and two applied voltages (zero voltage in the desorption step) in Figure 6.

Figure 6 also includes data on the so-called charge efficiency, or number of ions removed from the solution per electron transferred between the electrodes [24]. There is no significant difference between MCDI and SE-MCDI at low salt concentrations and low voltages, due to an almost optimum performance of the membranes. This makes the system reach the maximum efficiency value. However, at 100 mM and 1.2 V applied voltage, the membranes alone lose their efficiency due to the mentioned incomplete blocking of coions. The energetic cost of the removal of the ions from the main solution evolves inversely to efficiency, reaching higher values when the efficiency is lower (Figure 6).

**Figure 7:**
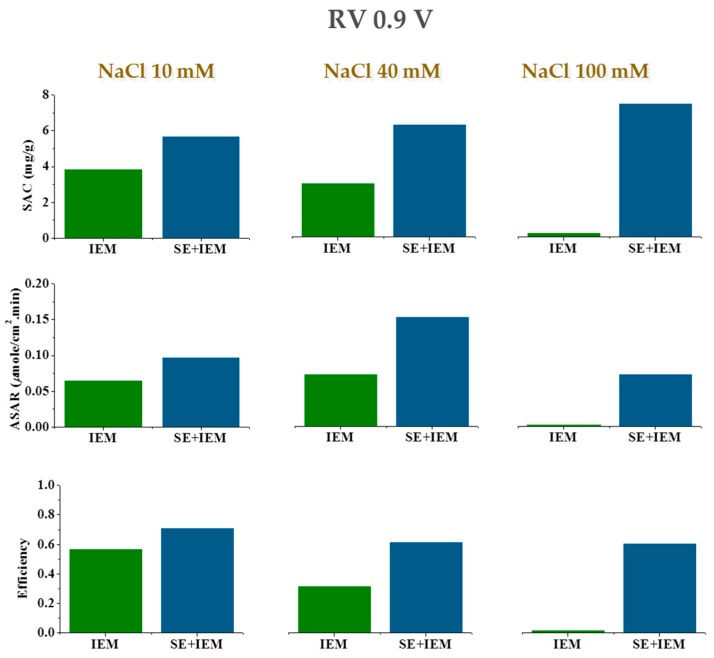
Salt adsorption capacity (*SAC*), adsorption rate (*ASAR*), and charge efficiency comparison between the use of membranes alone (MCDI) and the combination with polyelectrolyte coating (SE + MCDI).

Overall, a clear improvement is found when treated electrodes are compared to bare ones. Note that using SE-MCDI reduces the energy input needed to remove ions, mostly for higher voltages and higher salt concentrations. This result is better appreciated under RV desorption conditions, as clearly observed in Figure 7, where we include *SAC*, *ASAR*, and charge efficiency for the three sodium chloride concentrations. Note that the fact that the membranes do not fully reject coions reduces the performance of MCDI, as measured by both *SAC* and charge efficiency. In contrast, the combined SE + MCDI approach yield values of these quantities almost independent of the concentration. The differences are not so noticeable in the values of the adsorption rate, *ASAR*, since the cycle duration is longer when polyelectrolytes are used, if one attempts to maximize the amount of salt retained by the electrodes.

Jointly considered, these data show that the combined use of membranes and PE layers, even at moderately high saline concentration, constitutes a system that is capable of desalinating the solution with an almost perfect performance, opening the possibility of extending the applicability range of CDI techniques. The fact that we can partially desalinate 100 mM NaCl solutions appears very significant, as it is something not achievable when only membranes are used. The results can be explained with the theoretical model of the ion transport described below, considering the presence of the polyelectrolyte layer and the ion exchange membrane.

## 4. Theoretical Predictions

**Figure 8:**
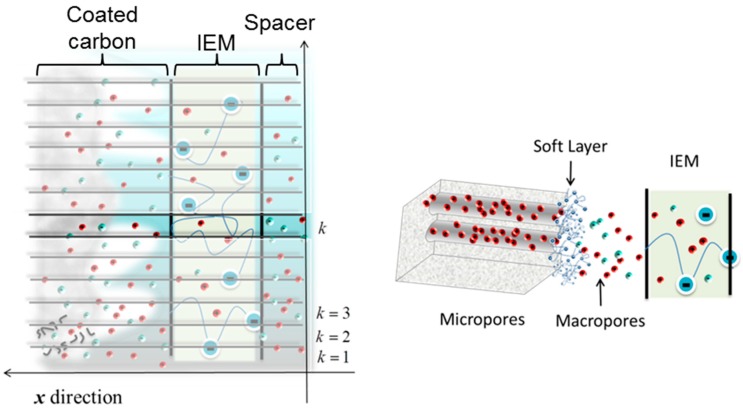
Sketch of half cell: One polyelectrolyte coated porous electrode in contact with a cation exchange membrane and the spacer channel. The flow of solution is from bottom to top.

The theoretical predictions are based on a modified membrane-CDI model [15], to take into account the presence of the polyelectrolyte layer. Based on the 1D model [25,26,27], it is possible to calculate the kinetics of ion adsorption. The model consists in dividing the cell into a certain number, *M*, of subcells, designed by *k* (k=1,…,M), along the spacer channel, as Figure 8 shows.

The objective is the evaluation of the flux of ions established between the spacer and the electrode in each subcell, so as to determine the salt concentration in it. This is performed by carrying out a mass balance in each of the regions. Note that the ionic motion also brings about an electrical current, and hence the condition must be fulfilled that the potential drop (with respect to the center of the channel) in each subcell must be equal to half the potential difference applied to the cell.

### 4.1. Mass Balance Equation

The mass balance equation for the total salt concentration is written for each region of the cell, namely spacer channel, ion exchange membrane, coated macropores, and micropores:

#### 4.1.1. Spacer Channel

The salt concentration in the *k*th subcell of the spacer channel changes with time due to the flux through the membrane, *j_k_*, in the *x* direction, and the convective transport of ions from the previous subcell, *k*−1, given, respectively, by the first and second terms in the right hand side of Equation (4):

(4) $$L_{sp}\frac{dc_{sp,k}}{dt}=-j_{k}+\frac{M\phi_{v}}{A_{sub}}\left(c_{sp,k-1}-c_{sp,k}\right)$$

where *L_sp_* is the spacer channel length, and *A_sub_* is the projected area of the electrodes. We call *c_sp,k_* the salt concentration in the *k*th subcell of the spacer, neglecting any variations in the *x* direction.

#### 4.1.2. Membranes

For highly charged membranes, the Nernst-Planck equation can be linearized and the flux through the membrane in the *k*th cell, *j_k_*, takes the following form [28,29]:

(5) $$j_{k}=-\frac{D}{L_{mem}}\left[\Delta c_{T,mem,k}-\rho_{mem}\Delta \psi_{mem,k}\right]$$

where *D* is the average diffusion coefficient of the two ionic species, *ρ_mem_* is the charge density of the membrane, and $\Delta c_{T,mem,k}$ is the difference of concentration between the membrane/spacer and membrane/macropore interfaces of the *k*th subcell:

(6) $$\Delta c_{T,mem,k}=\sqrt{\rho_{mem}^{2}+{\left(2c_{sp,k}\right)}^{2}}-\sqrt{\rho_{mem}^{2}+{\left(2c_{M,k}\right)}^{2}}$$

where *c_M,k_* is the salt concentration in the macropores. The potential difference across the membrane, $\Delta \psi_{mem,k}$, is linearly related to the electric current, *i_k_*, as:

(7) $$i_{k}=-\frac{D}{L_{mem}}\langle c_{T,mem,k}\rangle \Delta \psi_{mem,k}$$

where $\langle c_{T,mem,k}\rangle$ is the average concentration in the membrane. It must be noted that there is no salt accumulation in it, because the flux of ions is constant across it. Hence, the ionic current $i_{k}$ is due to the difference between counterion and coion fluxes.

#### 4.1.3. Electrodes

Finally, the balance of salt in the electrode is determined by the flux of ions through the membrane modifying the macropore, *c_M_*, and micropore, *c_μ_*, concentrations as:

(8) $$\frac{d}{dt}\left[2p_{M}c_{M,k}+p_{\mu }\left(c_{\mu ,k}^{+}+c_{\mu ,k}^{-}\right)\right]=\frac{j_{k}}{L_{e}}$$

where *p_M_* is the macroporosity, *p_|ì_*, the microporosity, *L_e_* is the thickness of the electrode, and $c_{\mu ,k}^{+}\left(c_{\mu ,k}^{-}\right)$ is the concentration of cations (anions) in the micropores of the *k*th subcell.

In the absence of the soft layer, the relationship between concentrations in macro- and micropores is given by the modified-Donnan model [30,31]. However, by considering the polymeric coating, the concentration outside the micropore corresponds to that of the “soft” layer, $c_{SE,k}^{}$, that is calculated assuming a Donnan balance inside (similar to the membrane):

(9) $$c_{SE,k}^{+}+c_{SE,k}^{-}=\sqrt{\rho_{pol}^{2}+{\left(2c_{M,k}\right)}^{2}}$$

where *ρ_pol_*is the charge density of the polyelectrolyte. The salt concentrations in micropores and soft layer are linked by the modified-Donnan model.

(10) $$c_{\mu ,k}^{+/-}=c_{SE,k}^{+/-}\exp(-z_{i}e\Delta \psi_{D,k}/k_{B}T)$$

where $\Delta \psi_{D,k}$ is the Donnan potential at the *k*th subcell, with respect to that in the soft layer. As the macropore is electrically neutral, the excess of charge in the micropores, $\rho_{\mu ,k}=z^{+}c_{\mu ,k}^{+}+z^{-}c_{\mu ,k}^{-}$, is determined by the current *i_k_*:

(11) $$L_{e}\frac{d}{dt}\left(p_{\mu }\rho_{\mu ,k}\right)=i_{k}$$

### 4.2. Boundary Conditions

Note that the current is invariant in the *x* direction, that is, the current across the spacer, the membranes, and their interfaces and that going into the micropores are all identical. In the spacer channel, a constant resistance is assumed in such a way that the current is proportional to the potential decay:

(12) $$i_{k}=2Dc_{sp,k}\frac{\Delta \psi_{sp,k}}{L_{sp}}$$

Moreover, another boundary condition has to be fulfilled: Half the external voltage applied between electrodes, *V_cell_*, is the superposition of the potential drop over each part of the subcell:

(13) $$\frac{1}{2}V_{cell}={\left(\Delta \psi_{sp}+\Delta \psi_{memb/sp}+\Delta \psi_{memb}-\Delta \psi_{memb/elec}+\Delta \psi_{elec}+{\left(\Delta \psi_{d}+\Delta \psi_{St}\right)}_{\mu }\right)}_{k}$$

where $\Delta \psi_{St}$ is the Stern layer potential at the micropores. In this simplified approach, the potential drop across the porous electrode, $\Delta \psi_{elec}$, and the spacer, $\Delta \psi_{sp}$ (Equation (12)), is characterized by an electrical resistance linearly dependent on macropore and spacer channel salt concentration, respectively.

**Figure 9:**
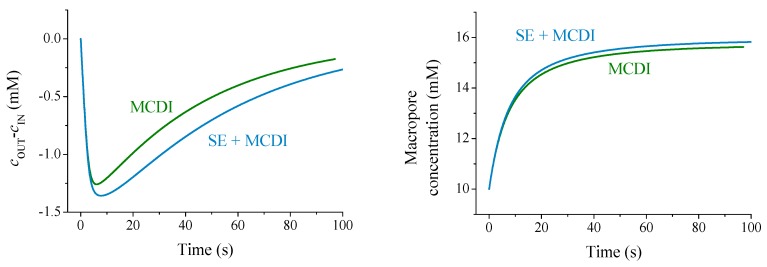
Theoretical predictions of concentration difference (**left**) and macropore concentrations (**right**) comparing MCDI and SE + MCDI. The quantities used for the calculation are: Porosities $p_{\mu }=0.2\;(\mathrm{micro}),\;p_{M}=0.3\;(\mathrm{macro})$; stern capacitance $C_{St}=10^{8}\;{F/m}^{3}$; dimensions of, respectively, membrane, spacer and electrode: $L_{mem}=140\;\mu m$, $L_{sp}=500\;\mu m$, $L_{e}=300\;\mu m$; Charge densities of membrane and polyelectrolyte: $5\times 10^{3}\;{\mathrm{mol}/m}^{3}$, and 10^3^ mol/m^3^.

After integrating the equations (using a MATLAB^®^ routine), we can analyze the theoretical predictions for the experimental conditions used in this work in order to compare adsorption curves using either ion exchange membranes or the polyelectrolyte coating in combination with ion exchange membranes. The predicted concentration differences as a function of time during the adsorption stage are represented in Figure 9.

The trend of theoretical predictions for the solution concentration difference agrees with the experimental results (Figure 3), although reasonable differences are found due to the large number of parameters to be fitted. The theory is in line with the hypothesis that an excess of polymer concentration (both membrane and soft layer) blocks coions in the macropore causing an enhancement of adsorption (see the increment of ionic concentration in the macropores during the adsorption step in Figure 9). Therefore, the polyelectrolyte layer favors the overall salt adsorption, due to the contributions of macropore reservoirs.

## 5. Conclusions

The desalination process known as capacitive deionization (CDI) is based on the high porosity of activated carbon materials and their subsequent capacity for absorbing comparatively large amounts of ions when they are electrically charged by establishing a potential difference between two opposing electrodes. When the technique is used with untreated electrodes (standard CDI), salt-adsorption capacity (*SAC*) values are moderate (about 3.5 mg/g in the best case, with a charge efficiency around 50%). The progress accompanying the use of ion exchange membranes (MCDI), or of polymeric coatings (SE-CDI) is clear and agrees with reported results, *SAC* values reaching 7–10 mg/g.

The main original contribution of this work is the use of a combination of membranes and polyelectrolyte coatings. The improvement is clear in all cases, as manifested by the superior values of *SAC* and charge efficiency, as well as the faster kinetics of the process. It also appears very significant that one can partially desalinate 100 mM NaCl solutions, something not achievable when only membranes are used. The results are explained by a theoretical model of the ion transport in this process considering the presence of the polyelectrolyte layer and the ion exchange membrane. The model explains the advantage of the combined method by the fact that the polyelectrolyte and membrane together enhance the accumulation of coions in the macropores (by blocking their expulsion into the spacer) while favoring the entrance of counterions, thus increasing the neutral salt adsorption in the macropores and the desalination of the solution flowing along the spacer. In summary, these results open the possibility of extending the range of applicability of CDI techniques.

## Supplementary Materials

The following are available online at https://www.mdpi.com/2073-4360/11/10/1556/s1, Figure S1: Relative solution conductivity and current intensity, for bare carbon electrodes and MCDI while applying 1.2 V at ZV and RV in the desorption step.
