# Supplementary material for: Assembly of Soft Electrodes and Ion Exchange Membranes for Capacitive Deionization
Source: Polymers (Basel). 2019 Sep 25;11(10):1556. doi: 10.3390/polym11101556 (PMC6836081; doi:10.3390/polym11101556)
Supplement: Supplementary file 1 [file polymers-11-01556-s001.pdf]

polymers

MDPI

Supplementary Materials: Assembly of Soft Electrodes and Ion Exchange Membranes for Capacitive Deionization

Silvia Ahualli [1,*], Sergio Orozco-Barrera [1], María del Mar Fernández [2], Ángel V. Delgado [1] and Guillermo R. Iglesias [1,*]

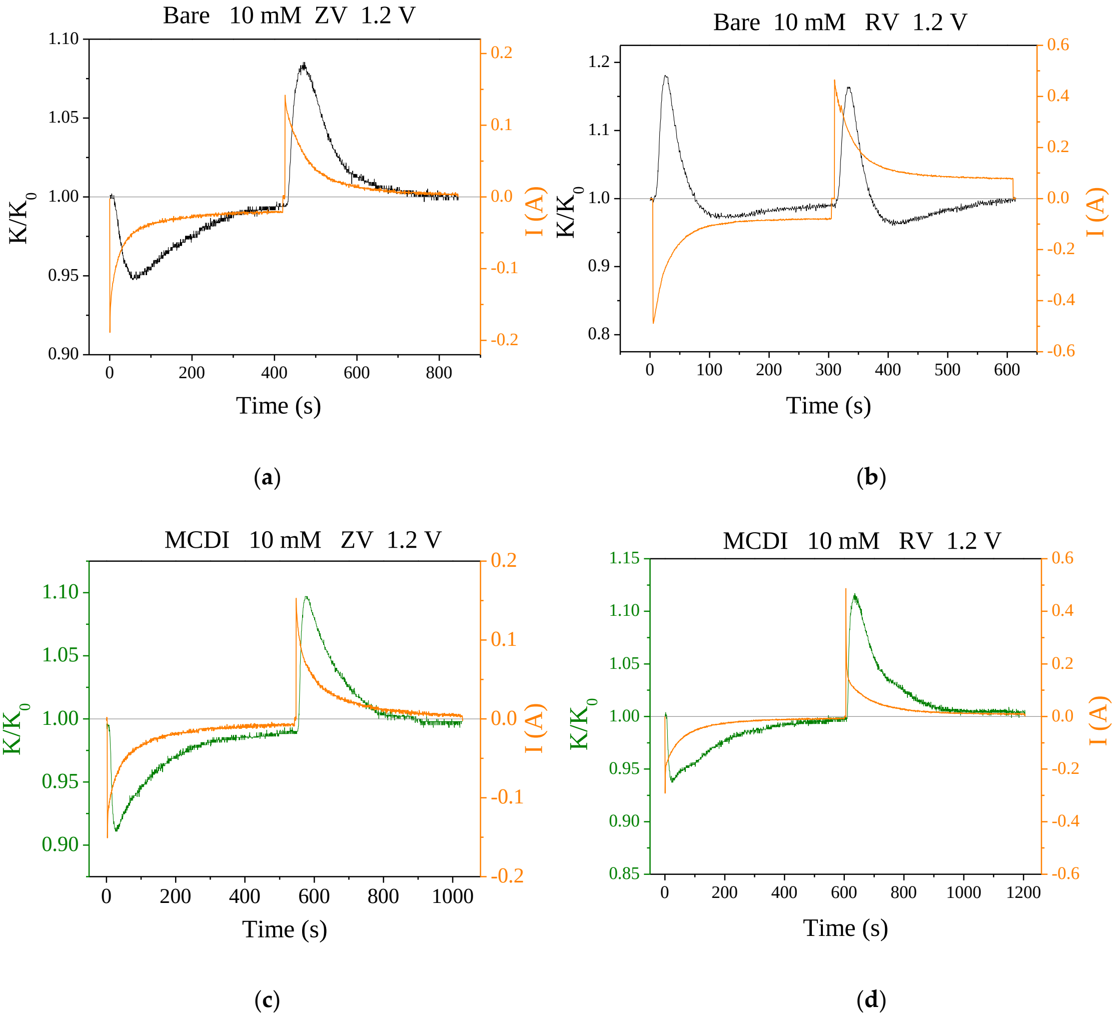

Figure 1. Exit solution conductivity, *K*, relative to that of the 10 mM NaCl feed solution, K_0, and current intensity, for bare carbon electrodes (top) and MCDI (bottom) while applying 1.2 V at ZV (**a** and **c**) and RV (**b** and **d**) in the desorption step.

© 2019 by the authors. Submitted for possible open access publication under the terms and conditions of the Creative Commons Attribution (CC BY) license (http://creativecommons.org/licenses/by/4.0/).
